# Vaccination for adults and their children: insights from survey and experimental data

**DOI:** 10.1186/s13561-025-00685-w

**Published:** 2025-10-24

**Authors:** Yiting Guo, Yan Peng, Lijia Wei

**Affiliations:** 1 Economics and Management School, Wuhan University, Wuhan, China; 2 College of Economics and Management, Huazhong Agricultural University, Wuhan, China

**Keywords:** Vaccination, Risk preference, Prosocial preference, Omission bias, I10, I12, C91

## Abstract

Vaccines are widely acknowledged as one of the most efficient and effective strategies for preventing diseases. This study, based on two waves of public survey data and complemented by incentivized experiments, investigates how behavioral preferences shape actual decisions to receive COVID-19 vaccinations. By combining these methods, the research aims to enhance both internal and external validity. Our findings indicate that individuals with elevated levels of prosociality show a greater propensity to receive the initial dose of the vaccine, a pattern that holds across both the general public and university students. In contrast, parental decisions regarding child vaccination are significantly associated with greater risk taking and lower omission bias, but not with prosocial preferences.

**Supplementary Information:**

The online version contains supplementary material available at 10.1186/s13561-025-00685-w.

## Introduction

The topic of vaccination has been extensively investigated within the behavioral economics literature [1, 2]. However, there remains a notable gap in examining the similarities and differences in decision-making processes within households regarding adult and child vaccinations. A key challenge lies in the fact that vaccines for adults and children are typically not of the same type, which complicates efforts to conduct empirical research or field experiments within a unified framework. Additionally, while adults make decisions regarding their own vaccinations, the decision-making process for children’s vaccinations is usually undertaken by their parents. The global COVID-19 pandemic, however, has made it possible to observe vaccination decisions for adults and children who receive the same type of vaccine. This unique context raises critical questions: Are the decision-making processes for adult and child vaccinations within a household aligned, and what behavioral factors drive these decisions? This paper provides a comparative analysis of adult vaccination behavior and parental decision-making regarding childhood vaccination. To empirically test our hypotheses, we use data from two waves of public online surveys, which include information on adult preferences, vaccination decisions for both adults and children, as well as a supplementary student sample capturing college students’ preferences and vaccination behavior.

Vaccination remains one of the most cost-effective and impactful methods for infectious disease prevention, averting an estimated 4 to 5 million deaths annually from diseases such as diphtheria, tetanus, pertussis, measles, and influenza. Expanding immunization coverage globally could potentially save an additional 1.5 million lives annually (WHO, 2019)^1^. Despite its proven effectiveness, vaccine hesitancy—defined as the delay in acceptance or refusal of vaccines despite their availability—poses a significant challenge to achieving optimal immunization rates. Vaccine hesitancy has been recognized as a critical public health issue that undermines efforts to achieve herd immunity and hinders epidemic control, even prior to the COVID-19 pandemic (WHO, 2019)^2^.

Behavioral economics has contributed significantly to understanding vaccination behavior by examining both individual-level and contextual determinants of vaccine hesitancy. Dubé et al. [3] emphasized that variations in diseases and their corresponding vaccines create unique behavioral factors influencing individual decision-making about vaccination. Dubé et al. [4] expanded on this by employing a social-ecological model to summarize factors affecting vaccination behavior, including individual, interpersonal, organizational, community, and public policy dimensions. Childhood vaccination decisions, distinct from adult vaccination decisions, are often influenced by parental perceptions of risks and benefits, leading to different decision-making dynamics. Previous research has indicated that vaccine hesitancy is more prevalent for childhood vaccinations compared to adult vaccinations [5].

This study explores several behavioral factors that might influence vaccination choices: prosocial preference, risk preference, and omission bias. Firstly, because COVID-19 is highly contagious, individual vaccination decisions affect not only personal health but also community transmission. Vaccination helps build herd immunity and protect vulnerable populations, making prosocial preference—the willingness to act for the benefit of others—a potentially important driver of vaccination uptake. Secondly, risk preferences are key to vaccination choices because of uncertainties about infection risks and potential side effects. Lastly, omission bias, which is a tendency to prefer negative outcomes from not acting rather than from acting, might cause individuals, especially parents, to avoid vaccinating their children due to perceived risks, even if the overall harm from not vaccinating is greater. Understanding these behavioral factors can offer vital insight into crafting effective strategies to increase vaccination coverage.

To investigate these factors, we begin by reviewing the relevant literature and developing hypotheses concerning how adults make vaccination decisions for themselves or their children, based on prosocial preferences, risk preferences, and omission bias. Guided by these hypotheses, we conducted two waves of public surveys between 2020 and 2022, collecting data from a total of 1,807 valid responses^3^. Wave 1 focused on adult vaccination behaviors, post-vaccination protective measures, and general behavioral preferences. Wave 2 gathered information on childhood vaccination decisions, examining how parental behavioral preferences influenced their decisions for their children’s vaccinations. As a robustness check for the adult vaccination analysis (Wave 1), we additionally draw on data from a student sample surveyed shortly after the first centralized vaccination campaign organized by Wuhan University in 2021, which recorded individuals’ initial vaccine doses. A summary of the two public survey waves and the Student Sample, including key information on data collection and task types, is provided in Table A1 of Appendix A in Supplementary Material.

We integrated online surveys with incentive-compatible online experiments to enhance both the internal and external validity of our empirical analysis. The online surveys facilitated large-scale data collection across a broad geographic scope and enabled us to target key populations, such as families with children—thereby strengthening external validity through a more diverse and representative sample. Complementing this, we conducted online experiments with college students as a robustness check, using incentivized preference data that had been collected prior to the actual vaccination rollout, and subsequently tracked their vaccination decisions. This temporal sequencing helps mitigate concerns about reverse causality—specifically, the possibility that vaccination behavior might influence reported preferences—thus bolstering internal validity. By combining these two approaches, we leverage their complementary strengths to enhance the overall validity of our findings. Moreover, our empirical strategy provides a consistent lens through which to interpret behavioral patterns in both adults’ vaccination decisions and parental choices for their children.

This paper makes several novel contributions to the literature on vaccination behavior. First, unlike most previous studies focusing solely on vaccination willingness, this research utilizes actual vaccination decisions as the key outcome variable, providing more accurate insights into behavioral patterns. By drawing on diverse samples, including the general public^4^ and university students, this study also ensures greater external validity. Second, given that the COVID-19 vaccine in China was administered free of charge, and vaccination campaigns were highly coordinated, the impact of traditional barriers such as cost and access was minimized. This unique setting allows for an examination of core behavioral factors without the confounding effects of cost and accessibility. Third, this research leverages the unique context of COVID-19, in which parents need to make decisions for both their own vaccination as well as that of their children. By examining adult and childhood vaccination decisions within a unified empirical framework, this study overcomes a significant limitation in prior research that often examined these groups separately.

The paper is structured as follows: Literature review and hypotheses section presents the literature review and hypotheses. The first wave: adult behavioral preferences and own vaccination and The second wave: parental behavioral preferences and child vaccination sections respectively present the empirical analyses of adult and child vaccination decisions, based on two waves of public online survey data. The penultimate section conducts robustness checks to assess the reliability of the results. The final section concludes with a summary of the findings, policy implications, and directions for future research.

## Literature review and hypotheses

### Adult vaccination

Behavioral preferences have increasingly been recognized as important determinants of adult vaccination behavior, especially in the context of novel and uncertain health risks such as COVID-19. Among these preferences, prosocial motivations and individual attitudes toward risk have received particular attention. Prosocial individuals may be more inclined to vaccinate in order to protect others, while individuals with lower risk tolerance may experience heightened concerns about either vaccine side effects or the risks of infection, which can further influence their vaccination decisions. These behavioral tendencies can shape individual vaccination decisions beyond demographic or informational factors. We therefore propose the following overarching hypothesis:

- Hypothesis 1 (Adult behavioral preferences and vaccination):
  Behavioral preferences influence whether adults choose to vaccinate themselves.

In what follows, we review existing evidence and formulate hypotheses regarding the influence of prosocial preferences and risk preferences on adult vaccination decisions.

Two main streams of research have examined the role of prosociality in vaccination. We begin with studies on prosocial information interventions, which have received extensive attention in public health communication, and then turn to studies that examine the role of prosocial preferences, which provide complementary theoretical foundations.

The first line of research investigates prosocial information interventions, examining how messages that highlight social benefits influence individuals’ vaccination decisions. For example, Betsch et al. [6] found that populations in countries with collectivist cultural values exhibit a higher willingness to vaccinate, suggesting that social norms and cultural context significantly influence vaccination behavior. Mussio et al. [7] showed that campaign messages emphasizing social benefits effectively increased influenza vaccination rates among young individuals through the field experiments conducted in the US. At the same time, other studies caution that the effects of such messages can be heterogeneous or even counterproductive. For instance, Galizzi et al. [8] demonstrated that providing vaccination coverage information could crowd out conformity effects, resulting in free-riding when coverage is already high. Taken together, these studies highlight the practical importance of prosocial information in mobilizing vaccination, while also revealing that its impact depends on context, framing, and population heterogeneity.

The second line of research takes a different approach by examining prosocial preferences as individual traits and investigating their relationship with vaccination behavior. Shim et al. [9] used survey data on actual influenza vaccination combined with game-theoretic epidemiological modeling to show that individuals reporting stronger altruistic concern for others were more likely to vaccinate, bringing private decisions closer to the social optimum. Vietri et al. [10] used scenario-based surveys and found that willingness to vaccinate increased when vaccination primarily benefited others, consistent with altruistic motivations. Experimental approaches provide complementary evidence. In an interactive vaccination game, Böhm et al. [11] measured participants’ social value orientation and found that those with stronger other-regarding preferences were more willing to vaccinate, even when self-interest alone would suggest free-riding. Campos-Mercade et al. [12] employed incentivized economic tasks to elicit prosociality and showed that more prosocial individuals reported greater engagement in—and intentions to adopt—COVID-19 protective behaviors, including vaccination. Together, this body of work provides a behavioral foundation: directly measured prosocial preferences are robust predictors of vaccination intentions and, in some cases, actual uptake.

Our study aligns more closely with this second line of research, as we directly measure prosocial preferences to assess their impact on vaccination behavior. Building on this cumulative evidence—especially findings that highlight the role of prosocial preferences in shaping vaccination behavior—we propose the following hypothesis:

- Hypothesis 1a (Prosocial preferences and adult vaccination):
  Adults with stronger prosocial preferences are more likely to vaccinate themselves.

In addition to prosocial motivations, individual risk preferences have also been widely examined in relation to adult vaccination behavior. The relationship between risk preference and adult vaccination behavior or willingness remains an area of significant ambiguity within the literature. For instance, while Tsutsui et al. [13] found in Japan that more risk-averse individuals—those more concerned about contracting influenza—were more likely to vaccinate, Trueblood et al. [14] reported opposite results in the United States, where individuals with higher financial risk tolerance were more inclined to take both novel (COVID-19) and established (flu) vaccines. Binder et al. [15] further documented that the association between risk preference and vaccination willingness can vary by gender and decision context, highlighting the role of subgroup heterogeneity in shaping observed outcomes.

Both theoretical modeling and empirical work suggest that these inconsistencies are primarily driven by two factors: (i) the inherent dual-risk nature of vaccination—balancing infection risk against side-effect risk—and (ii) differences in how risk preferences are elicited. Regarding the first factor, Tsutsui et al. [13] explicitly examined how individuals weigh the trade-off between infection risk and potential side effects when deciding whether to vaccinate. In line with this perspective, Crainich et al. [16] formally model the dual-risk structure and demonstrate that the effect of risk aversion on vaccine uptake depends on which risk dominates in perception. Regarding the second factor, Massin et al. [17] show that lottery-type risk tasks are better predictors of vaccination behavior than subjective self-assessments, underscoring the importance of measurement approaches in shaping observed associations.

Overall, the literature does not support a consistent directional prediction: the association between risk preferences and adult vaccination varies with contextual factors such as vaccine type, perceived salience of different risks, and risk-elicitation methods. Given this ambiguity—especially in the case of COVID-19—we propose the following non-directional exploratory hypothesis:

- Hypothesis 1b (Risk taking and adult vaccination):
  Adults’ risk-taking tendencies are associated with their vaccination decisions.

### Children vaccination

The influence of parental behavioral preferences on children’s health decisions is a significant topic at the intersection of health and behavioral economics. In the context of adult vaccination, individuals make decisions autonomously, whereas for childhood vaccination, parents or guardians are responsible for these choices. Consequently, parental behavioral preferences play a pivotal role in vaccination decisions. We therefore propose the following overarching hypothesis:

- Hypothesis 2 (Parental behavioral preferences and children vaccination):
  Parental behavioral preferences influence whether they choose to vaccinate their children.

Among the behavioral factors shaping parental vaccination decisions for their children, prosocial preference—the willingness to incur personal cost to benefit others—has received increasing attention. Quadri-Sheriff et al. [18] conducted a systematic literature review to assess whether altruistic motivations—specifically, the desire to confer benefits to others—affect parents’ decisions to vaccinate their children. Although some evidence indicates that prosocial preference plays a role, the degree to which prosocial considerations systematically impact vaccination behavior remains ambiguous. For instance, Sobo [19] found that only 11.3% of parents explicitly linked herd immunity to their decision to vaccinate, suggesting that the broader social benefits of vaccination may not be a salient factor for most parents. Furthermore, Amin et al. [20] reported that parental vaccine hesitancy is also related to moral beliefs, suggesting that prosocial considerations may be overridden by other normative and moral frameworks. These findings indicate that while prosocial motives can influence vaccination behavior, they are often secondary to other behavioral factors, such as risk perceptions and moral attitudes.

Given the mixed evidence, it is important to empirically test whether and how prosocial preferences are associated with childhood vaccination behavior. We therefore propose the following non-directional hypothesis:

- Hypothesis 2a (Prosocial preferences and children vaccination):
   Parents’ prosocial preferences are associated with their decisions to vaccinate their children.

Alongside prosocial motivations, scholars have also examined whether parents’ risk attitudes influence their decisions to vaccinate their children. Tang et al. [21] shows that participants were more willing to vaccinate their children than themselves, suggesting that parents may be more sensitive to risk when making decisions for their children compared to themselves. However, the risk in Tang et al. [21] specifically refers to the risk of death from non-vaccination, overlooking another important aspect of real-world risk—the possibility of side effects associated with vaccination. Diza [22] finds minimal evidence of a relationship between parents’ risk aversion and their decisions regarding children vaccination, where the “risk” refers to the potential adverse effects of vaccination and the uncertainty surrounding the vaccination process.

Taken together, prior studies suggest that while parental risk preferences may influence childhood vaccination decisions, the evidence is mixed and does not support a consistent directional prediction. We therefore frame the following exploratory hypothesis in a non-directional manner:

- Hypothesis 2b (Risk taking and children vaccination):
  Parents’ risk-taking tendencies are associated with their decisions to vaccinate their children.

In addition to value-driven traits such as prosociality and risk preferences, cognitive biases—particularly omission bias—may represent another important psychological barrier to vaccination. A key behavioral factor identified in the literature contributing to childhood vaccine hesitancy is “omission bias.” This bias describes the tendency to prefer inaction opting not to vaccinate—even when the probability of adverse health outcomes due to non-vaccination equals or exceeds the potential risks associated with vaccination [23]. This preference for inaction is often driven by an asymmetric weighting of perceived risks, where potential harms from action (i.e., vaccination side effects) are overemphasized relative to the harms of inaction (i.e., contracting a vaccine-preventable disease). Asch et al. [24] empirically demonstrated that omission bias influences parental decisions regarding pertussis vaccination, and subsequent studies have confirmed this finding for other vaccines, including those by Brown et al. [25], Polman [26], Ritov et al. [27], and Böhm et al. [11]. These studies collectively underscore the role of cognitive biases in shaping parental health decisions, highlighting the importance of addressing psychological barriers to increase childhood vaccination rates.

These findings suggest that omission bias can lead parents to deviate from optimal health decisions—avoiding vaccination despite greater risks from inaction. To formally assess this relationship, we propose the following hypothesis:

- Hypothesis 2c (Omission bias and children vaccination):
  Parents with stronger omission bias are less likely to vaccinate their children.

In sum, childhood vaccination decisions are shaped by a complex interplay of behavioral drivers—including motivational traits like prosocial and risk preferences, as well as cognitive biases such as omission bias. Together, these factors provide a multi-dimensional lens through which parental hesitancy can be understood and addressed.

## The first wave: adult behavioral preferences and own vaccination

To validate the relationship between individual preferences and COVID-19 vaccination, a large-scale online survey of the general public’s COVID-19 vaccination status was conducted in China from May to June 2021^5^. In addition, this wave investigates whether individuals planned to reduce personal protective measures after vaccination and how such decisions relate to their risk and prosocial preferences. This allows for a clearer understanding of the actual health implications of vaccination behavior under the influence of individual preferences.

### Procedure

Data for this wave were collected between May 20 and June 2, 2021, through an online survey platform called “Wenjuanxing”^6^. Participants were selected randomly from four cities — Hefei, Wuhan, Zhengzhou, and Xi’an — using IP address localization^7^. A total of 1,268 valid responses were obtained, and each participant received a reward of 5 yuan for completing the survey. The entire data collection process was conducted anonymously through the survey company. To ensure data quality, trap questions were randomly embedded throughout the questionnaire to identify and exclude invalid responses^8^.

Of the four cities, Hefei was undergoing a localized COVID-19 outbreak at that time, whereas Wuhan had been hit hard by an outbreak during the initial phase of the pandemic. As a control city, Zhengzhou was situated roughly equidistant from Hefei and Wuhan. In contrast, Xi’an also functioned as a control city, having never faced a COVID-19 outbreak at that time and being located far from the outbreak epicenters. The primary elements of the extensive online questionnaire mainly included:

- Prosocial preference was assessed using four questions related to behaviors such as donating blood, volunteering, making donations and giving up one’s seat for others, with higher scores representing higher levels of prosociality [28]^9^.
- The participants’ preference towards risk was assessed using the domain-specific risk-taking scale (DOSPERT) [29], which included 13 hypothetical questions^10^. A higher score represented a greater level of risk-taking.
- Participants were asked if they had taken the initial dose of the COVID-19 vaccine. (response options: Yes; No)
- Participants were asked whether they planned to reduce personal protective measures after receiving the COVID-19 vaccine, such as reducing the frequency of wearing masks (response options: Yes; No; Not Sure).

### Data

Table 1 presents the main characteristics of the entire sample as well as those for each city, showing that there were no obvious differences in characteristics between cities^11^. Overall, 74% of respondents had already received the initial dose of the COVID-19 vaccine at the time of survey submission. The sample was composed of 52.52% male participants, with an average age of approximately 30 years^12^. A majority (54.81%) held a bachelor’s degree, and 2.05% reported working in the healthcare sector. Regarding income, 44.24% of respondents fell within the annual income bracket of 20,000 to 100,000 RMB. Additionally, 26.10% of participants indicated that they had travel plans in the near future. The questionnaire also inquired about the timing of receiving the initial dose of the vaccine.

**Table 1:** Summary statistics for four cities

	Hefei	Wuhan	Zhengzhou	Xi’an	Total
Received the initial dose of the vaccine (June 2, 2021)	84%	76%	69%	67%	74%
Prosocial preference (1–5)	3.99	3.82	3.93	3.89	3.91
Risk taking (1–5)	2.89	2.92	2.83	2.88	2.88
Age	32.37	28.57	30.92	30.58	30.62
Male (%)	57.63	41.64	55.56	55.24	52.52
Education (%)					
Didn’t attend high school	1.87	1.58	0.95	2.86	1.81
High school or similar	16.82	8.83	11.43	11.75	12.22
College	23.05	17.67	29.84	20.95	22.87
Bachelor’s degree	49.53	60.25	52.38	57.14	54.81
Master’s degree or above	8.72	11.67	5.4	7.3	8.28
Work in the healthcare industry (%)	3.12	1.89	1.59	1.59	2.05
Annual income (%)					
Below 20 K (20,000 RMB)	20.25	30.28	27.30	33.02	27.68
20 K to 100K	50.78	36.91	44.13	45.08	44.24
100 K to 300 K	24.61	30.91	26.35	20.63	25.63
300 K to 1 M	4.36	1.26	2.22	1.27	2.29
Above 1 M	0	0.63	0	0	0.16
CPC (Communist Party of China) membership (%)	30.53	24.61	26.98	24.13	26.58
Have travel plans in the near future (%)	20.87	31.86	24.13	27.62	26.10
Observations	321	317	315	315	1268

**Fig. 1:**
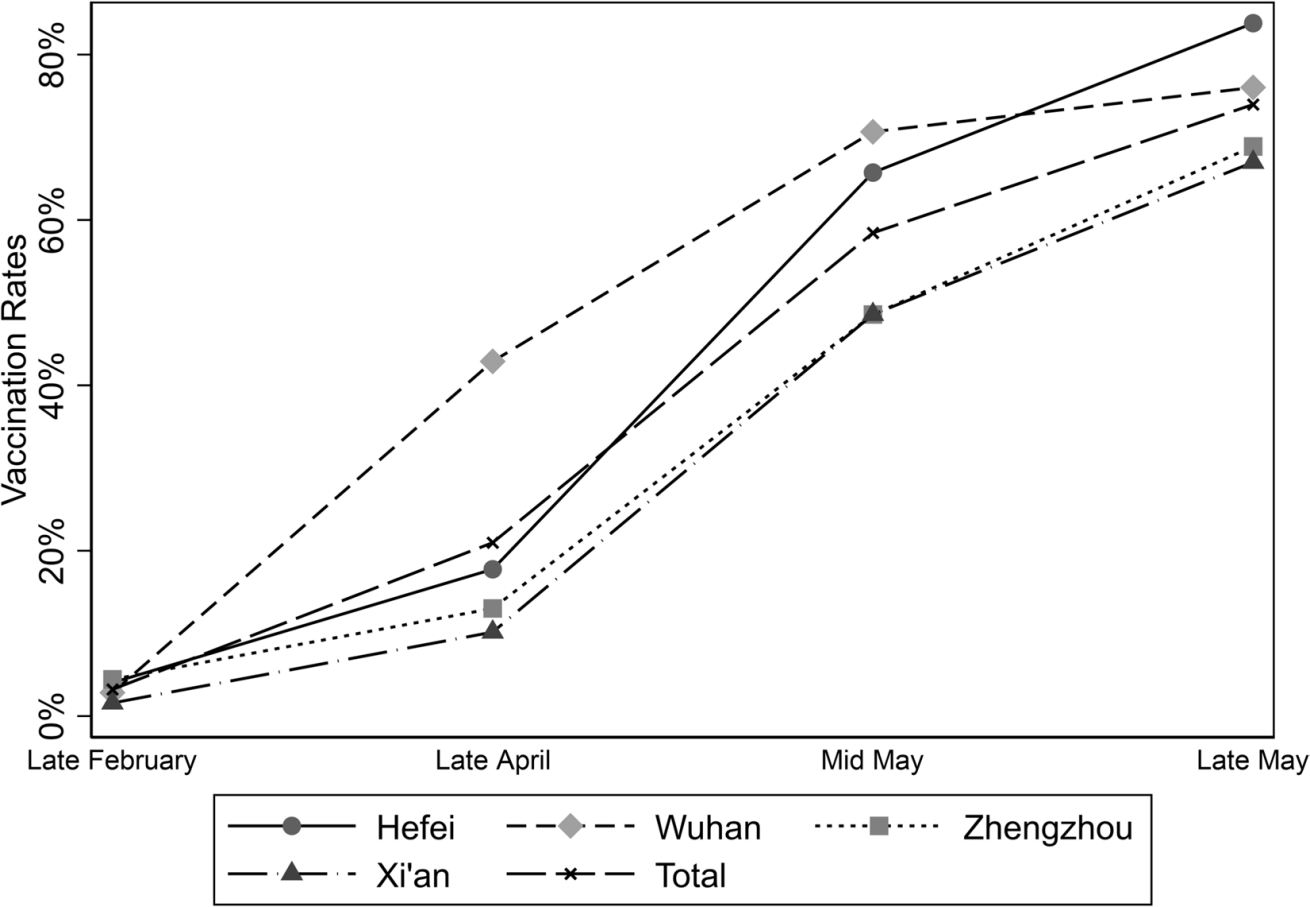
Vaccination rates in the samples from different cities (2021)

Figure 1 summarizes vaccination rates in different cities at various time points. As of late April 2021, Wuhan had a relatively high vaccination rate, while the rates in the other cities were below 20%. However, after a COVID-19 outbreak in Hefei in mid-May 2021, its vaccination rate increased significantly and surpassed that of Wuhan by late May, becoming the highest among the four cities. Conversely, Xi’an, which had not experienced a large-scale outbreak, had a relatively lower vaccination rate.

### Results

Table 2 compares differences in prosocial and risk preferences among participants from different cities. In terms of prosocial preference, vaccinated participants in all cities consistently demonstrated significantly higher levels of prosociality compared to their unvaccinated counterparts. Specifically, Hefei, Wuhan, and Xi’an showed significant differences at the 1% level, while Zhengzhou exhibited a slightly lower significance level at the 10% level. Overall, across all four cities, prosocial preference was significantly associated with vaccination status at the 1% level. In contrast, for risk taking, no significant differences were found between vaccinated and unvaccinated participants in most cities, except in Zhengzhou, where vaccinated participants exhibited greater risk taking, significant at the 10% level.

**Table 2:** Vaccination and adult preferences

	Not vaccinated	Vaccinated	*p*-value
*Panel A. Prosocial preference*			
Hefei	3.71	4.05	$<0.01$***
Wuhan	3.62	3.88	$<0.01$***
Xi’an	3.74	3.96	$<0.01$***
Zhengzhou	3.79	3.99	0.06*
*Panel B. Risk taking*			
Hefei	2.82	2.90	0.46
Wuhan	2.90	2.93	0.91
Xi’an	2.81	2.83	0.77
Zhengzhou	2.80	2.92	0.07*

Two-sided *p*-values based on Wilcoxon rank-sum test results are recorded in the last column. ***, **, and * indicate statistical significance at the 1%, 5%, and 10% levels, respectively

To control for the influence of other variables, Table 3 reports the average marginal effects estimated from logit models^13^. Model (1) controls for individual characteristics such as age, gender, education level, income, political affiliation (i.e., membership in the CPC), and whether the individual worked in the healthcare industry; Model (2) adds control for whether the individual planned to travel in the near future; Model (3) further includes city-level fixed effects. The dependent variable in each model is whether an individual had received the initial dose of the COVID-19 vaccine.

**Table 3:** Vaccination and behavioral preferences (adults)

Dependent variable	Received the initial dose of the COVID-19 vaccine				
Sample	All sample			Above 45 years	Above 55 years
	(1)	(2)	(3)	(4)	(5)
Prosocial preference	0.085***	0.084***	0.081***	0.118***	0.121*
	(0.016)	(0.016)	(0.016)	(0.035)	(0.063)
Risk taking	0.038	0.027	0.024	0.008	−0.064
	(0.024)	(0.024)	(0.024)	(0.043)	(0.068)
Control for characteristics	Yes	Yes	Yes	Yes	Yes
Have travel plans in the near future	No	Yes	Yes	Yes	Yes
Control city fixed effect	No	No	Yes	Yes	Yes
Control wave fixed effect				Yes	Yes
Model$\chi ^2$	64.233***	74.544***	99.039***	38.735***	29.029***
Observations	1268	1268	1268	264	70

1. This table reports marginal effects from the logit model, with robust standard errors in parentheses. ***, **, and * indicate statistical significance at the 1%, 5%, and 10% levels, respectively

2. Control for characteristics: Age, gender, education, work in the healthcare industry, annual income, CPC

3. While there are 73 observations for individuals aged 55 and above, only 70 observations are used in the logit regression. This is due to automatic exclusion of observations with perfect prediction or missing values by the logit model

4. Wave fixed effect: Columns (1)–(3) use data from wave 1. However, since columns (4) and (5) require sample filtering based on age, the wave 1 sample size is relatively small. Therefore, we also include samples from wave 2. Although Wave 2 was conducted later, we use self-reported vaccination dates to ensure that the outcome variable consistently reflects vaccination status as of May 2021. To account for potential differences arising from the use of different questionnaires, we control for questionnaire-fixed effects in the regression models for columns (4) and (5)

The results indicate that prosocial preference was significantly associated with vaccination behavior, with individuals exhibiting higher levels of prosociality being more likely to receive the COVID-19 vaccine. More specifically, a one-unit increase in prosocial preference is associated with an approximate 8 percentage point increase in the probability of being vaccinated. These findings are further confirmed in the robustness check using the college student sample and corroborate previous studies such as Shim et al. [9], which found that prosocial preferences are predictive of vaccination behavior. It also supports the theoretical prediction in Hypothesis 1a, which states that prosocial preferences increase the motivation for self-vaccination. By contrast, the coefficient on risk taking is positive but not statistically significant, a pattern that is also observed in the robustness check based on the college student sample. Thus, we do not find evidence supporting Hypothesis 1b, which posits an association between adults’ risk-taking tendencies and their vaccination decisions.

Furthermore, this study examined the influence of behavioral preferences on COVID-19 vaccination among older adults, specifically those aged 45 and older and those aged 55 and older^14^. The regression results in Models (4) and (5) indicate that prosocial factors significantly promote vaccination behavior among the middle-aged and elderly population, with a stronger effect observed compared to the full sample:^15^ compared to the 8.1% points increase observed in the full sample, a one-unit increase in prosocial preference raises the probability of being vaccinated by 11.8 and 12.1 percent points in the 45-and-older and 55-and-older subsamples, respectively.

**Fig. 2:**
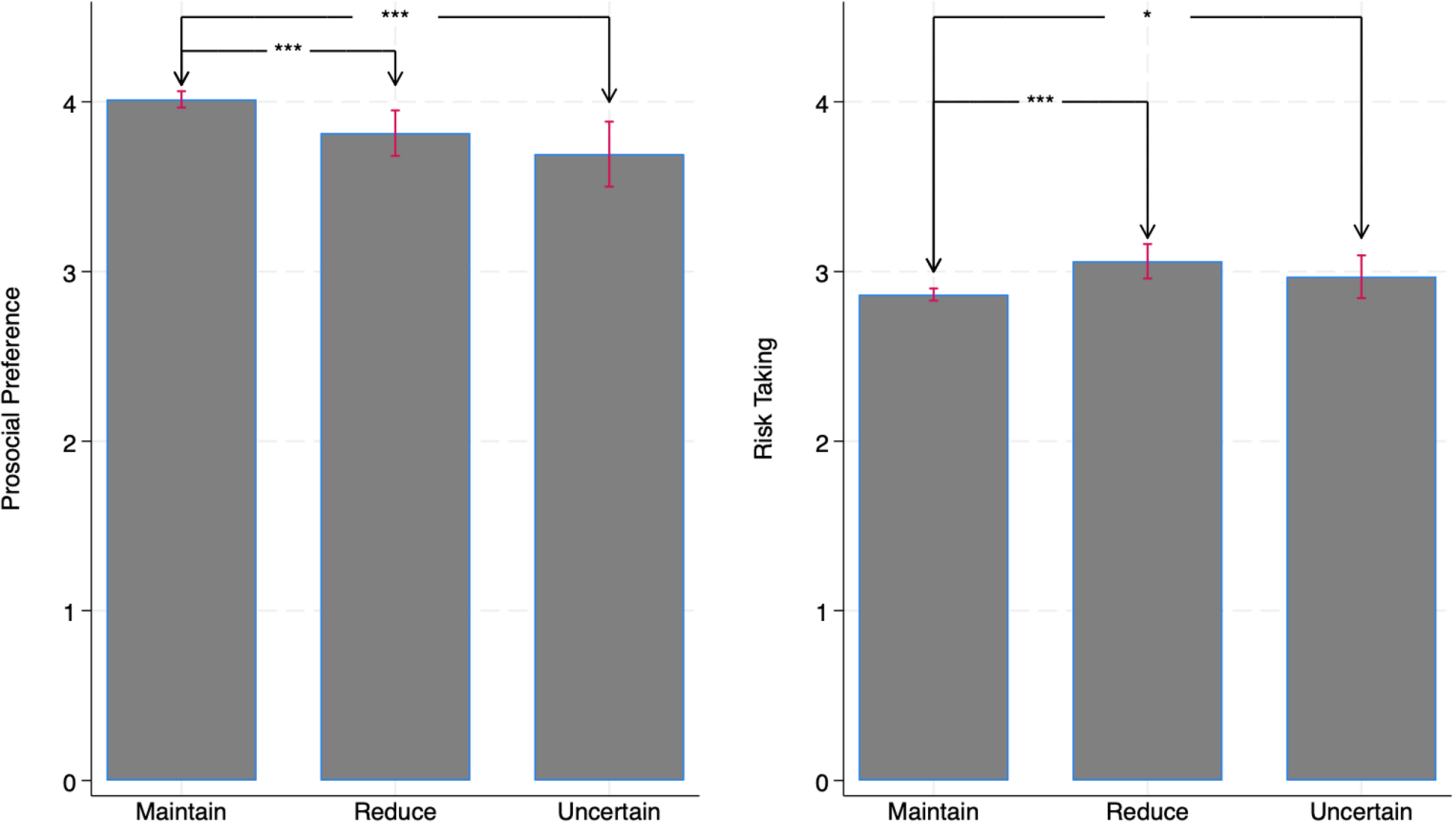
Whether subjects will reduce protective measures after vaccination. *Note:* The error bar represents the 95% confidence interval. ***, **, and * indicate statistical significance based on the Wilcoxon rank-sum test at the 1%, 5%, and 10% levels, respectively

Beyond the decision to vaccinate, we also examine whether behavioral preferences influence individuals’ willingness to maintain other preventive measures after vaccination. This helps assess whether the effects of prosociality and risk preferences extend to broader COVID-19 mitigation behaviors. To this end, we asked participants whether they planned to reduce protective actions (e.g., mask-wearing, distancing) after being vaccinated. Among respondents, 81.02% reported they would maintain such practices, 13.54% said they would reduce them, and 5.44% were uncertain—suggesting that vaccination does not substantially diminish protective vigilance. Moreover, individuals who intended to maintain protective behaviors exhibited higher prosocial preferences and lower risk-taking. This suggests that individuals with stronger prosocial preferences are more likely to continue preventive actions, whether through vaccination or measures like wearing masks. Conversely, those with greater levels of risk-taking are more inclined to ease personal protective measures after vaccination (Fig. 2).

## The second wave: parental behavioral preferences and child vaccination

The analyses presented in the first wave mainly focused on the role of behavioral preferences in adults’ decisions regarding their own COVID-19 vaccination. Given the critical role children play in achieving immunization goals for various diseases, understanding how parents make vaccination decisions for their children is essential. Literature suggests that parents’ decisions regarding their own vaccinations might differ significantly from decisions involving their children, warranting an examination of whether findings from adult vaccination studies extend to children’s vaccinations. This section aims to analyze the influence of parental prosocial preference, risk preference, and omission bias on their decisions to vaccinate their children.

### Procedure

Data collection in the second wave took place between December 11, 2021 and January 18, 2022, targeting participants from four cities — Wuhan, Zhengzhou, Xi’an, and Hefei — using the same survey platform. The initial participants were parents involved in the first wave of adult data collection, intending to assess the COVID-19 vaccination status of their children^16^. Focusing on subjects with children of the appropriate age resulted in some sample loss. The content of the children’s vaccination survey mainly included:

- The prosocial questions and DOSPERT risk preference questions were consistent with those in the first wave.
- Inquiries about the COVID-19 vaccination status of both the participants and their children.
- Three questions, derived from Asch et al. [24] and Polman [26], aim to explore “omission bias” in parents’ vaccination decisions for their children. Elevated scores signified a stronger omission bias, indicating a greater inclination to refrain from actively subjecting children to possible adverse vaccination effects^17^.

### Results

A total of 706 valid questionnaires were collected from participants with children aged 3 and above^18^. Since our study focuses on parental decisions regarding COVID-19 vaccination for eligible children, we restricted the sample to respondents who had at least one child aged 3 or older, in line with the official age eligibility for vaccination at the time of the survey. First, the characteristics of these children were analyzed both for the overall sample and by city. As presented in Table 4, the Kruskal–Wallis test indicates no statistically significant differences in the number, age, or gender distribution of children across the cities. On average, each family had approximately 1.37 children aged 3 and above, with an average age of 11 years and a roughly equal gender ratio.

**Table 4:** Data summary of children in each city

	Hefei	Wuhan	Zhengzhou	Xi’an	Total	$\chi ^2$	$p-value$
The number of children	1.39	1.33	1.50	1.27	1.37	4.06	0.25
	(0.57)	(0.56)	(0.64)	(0.47)	(0.57)		
Age	11.33	10.44	10.00	12.09	10.92	5.13	0.16
	(8.14)	(7.33)	(7.15)	(8.97)	(7.91)		
Male (%)	49.12	52.29	53.39	57.35	52.92	2.99	0.39
	(50.10)	(50.05)	(50.00)	(49.58)	(49.94)		
All child vaccinated (age $\ge 3$) (%)	82.86	82.47	81.29	82.53	82.29	0.17	0.98
	(37.80)	(38.12)	(38.09)	(39.12)	(38.20)		
Parents have been vaccinated (%)	97.71	97.94	97.67	96.99	97.59	0.00	1.00
	(14.99)	(14.25)	(15.16)	(17.14)	(15.34)		
Parent vs. Child Vaccination (Wilcoxon tests)	***	***	***	***	***		
Observations	175	194	171	166	706		

Standard errors are reported in parentheses; We performed balance tests for each variable across the four cities using the Kruskal-Wallis test. The $\chi ^2$ with ties and *p*-value are reported in the last two columns

In addition to basic demographic characteristics, Table 4 also reports the vaccination rates of children and their parents. The variable “All child vaccinated (age $\ge 3$)” equals 1 if all children aged 3 and above within the household have received the vaccine; otherwise, it equals 0.^19^ As shown in the fourth and fifth rows, the proportion of vaccinated parents is consistently higher than that of vaccinated children across all cities. Specifically, while 82.29% of households reported that all children aged 3 and above had been vaccinated, the corresponding rate for parents was 97.59%. To formally test whether this difference is statistically significant, we performed Wilcoxon signed-rank tests comparing paired vaccination outcomes within families. The sixth row of Table 4 reports the test results. In all four cities and in the full sample, the differences are highly significant (all *p*-values $< 0.001$), indicating that parents are significantly more likely to vaccinate themselves than to vaccinate their children.

Table 5 examines the relationship between parents’ behavioral preferences and their decisions to vaccinate their children. The data indicate that, unlike the findings for adult vaccination, parents’ prosocial preference did not differ significantly between those who had vaccinated their children and those who had not, suggesting no support for Hypothesis 2a. However, risk-taking was significantly associated with parental vaccination decisions, indicating support for Hypothesis 2b. Specifically, we find that parents with higher risk-taking tendencies are more willing to vaccinate their children. Furthermore, parents whose children were not vaccinated exhibited significantly higher omission bias compared to those whose children were vaccinated, providing support for Hypothesis 2c^20^.

**Table 5:** Parental behavioral preferences and children vaccination

	Not all children vaccinated (age) (*n*=125)	All child vaccinated (age) (*n*=581)	*p*-value
Prosocial preference	3.95	4.06	0.14
	(0.72)	(0.72)	
Risk taking	2.85	2.93	0.07*
	(0.50)	(0.54)	
Omission bias	3.43	2.91	$<0.01$***
	(0.86)	(0.89)	

Standard errors are reported in parentheses; Two-sided *p*-values based on Wilcoxon rank-sum test results are recorded in the last column. ***, **, and * indicate statistical significance at the 1%, 5%, and 10% levels, respectively

The descriptive analysis demonstrates that parents’ risk taking and omission bias significantly influence their decision to vaccinate their children. To better understand these relationships, the study performed Logit regressions, controlling for individual factors such as education level, annual income, healthcare profession, and CPC membership^21^. The regressions sought to uncover the behavioral understanding of parents’ vaccination choices by utilizing prosocial preference, risk taking, and omission bias as explanatory factors^22^.

The marginal effects reported in Table 6 show that risk taking significantly influences parents’ likelihood of vaccinating their children, while prosocial preference does not play a significant role—contrary to findings on adult vaccination behavior. Specifically, the results from Model (3) suggest that a one-unit increase in individual risk taking increases the likelihood of vaccinating one’s child by 7 percentage points. This finding addresses a gap highlighted by Quadri-Sheriff et al. [18], where the relative importance of prosocial preference as motivation in vaccination decisions was unclear. The results suggest that, while prosocial preference may be an important driver of individuals’ decisions to vaccinate themselves, it plays a less significant role in parental decisions about their children, not supporting Hypothesis 2a. Instead, parents’ risk-taking tendencies appear to be a crucial factor in these decisions, consistent with Hypothesis 2b. Our results show that parents with higher risk-taking tendencies are more willing to vaccinate their children.

**Table 6:** Parental behavioral preferences and children vaccination

Dependent variable	All child vaccinated (age $\ge 3$)		
	(1)	(2)	(3)
Prosocial preference	0.012	−0.005	−0.006
	(0.019)	(0.020)	(0.020)
Risk taking	0.065**	0.070**	0.070**
	(0.031)	(0.031)	(0.030)
Omission bias		−0.082***	−0.082***
		(0.015)	(0.015)
Control for characteristics	Yes	Yes	Yes
Control wave fixed effect	Yes	Yes	Yes
Control city fixed effect	No	No	Yes
Model $\chi ^2$	34.392***	50.547***	53.298***
Observations	706	706	706

1. This table reports marginal effects from the logit model, with robust standard errors in parentheses. ***, **, and * indicate statistical significance at the 1%, 5%, and 10% levels, respectively

2. Control for characteristics: Age, gender, education, work in the healthcare industry, annual income, CPC

3. Wave fixed effect: The analysis includes both follow-up respondents from Wave 1 and newly recruited participants in Wave 2; wave fixed effects are included to account for systematic differences across waves

Even after controlling for individual heterogeneity, omission bias remains a significant negative determinant of parents’ decisions to vaccinate their children in both Models (2) and (3), supporting the Hypothesis 2c. Specifically, the estimates from both models indicate that a one-unit increase in omission bias is associated with an 8.2 percentage point decrease in the probability of vaccinating one’s child, statistically significant at the 1% level. This finding highlights the persistent impact of omission bias on parental vaccination decisions, suggesting that parents are more hesitant to expose their children to perceived risks from vaccination than to protect them from potential harm through inaction^23^.

## Robustness checks

### Adult vaccination among college students: incentivized vs. non-incentivized measurement

The main empirical analysis based on public survey data focuses on adult and parent–child vaccination decisions in Wave 1 and Wave 2, whereas this section introduces an auxiliary dataset—the Student Sample—as a robustness check specifically for the adult vaccination results in Wave 1. This dataset contains incentivized, experimentally elicited measures of behavioral preferences, offering a contrast to the self-reported measures used in the main public survey. These behavioral preferences were collected in an earlier, independent experimental study and linked to student vaccination records gathered several months later, specifically for this research, allowing us to reduce the risk of reverse causality. To validate the comparability of behavioral preference measures across public and student samples, we conducted a supplementary correlation analysis using newly collected data with both incentivized and non-incentivized tasks. See Appendix C.3 in Supplementary Material for details.

The Student Sample enables us to test whether behavioral preferences, as measured in a controlled experimental environment, can reliably predict real-world vaccination behavior, thereby strengthening the internal validity of our main findings and corroborating the central conclusion from Wave 1.

#### Procedure

**Fig. 3:**
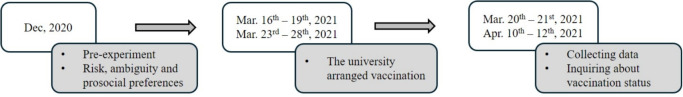
Experimental timeline

The Student Sample participated in individual preference experiments conducted in December 2020. Data on college student vaccinations were collected at Wuhan University between March and April 2021, providing a unique opportunity to link individual behavioral preferences with subsequent health-related decisions. Specifically, the Student Sample data collection incorporated prior measures of students’ risk, ambiguity, and prosocial preferences [30]. Wuhan University was among the first institutions in China to promote COVID-19 vaccination, allowing vaccination behavior to be studied in an environment of high uncertainty and limited prior information. Following the first round of vaccinations, a vaccination questionnaire was administered and collected within one month (Fig. 3)^24^.

We first introduce the individual preference data, which constitute the first of two main data components used in this robustness check. The decision tasks conducted in December 2020 were designed to gather data on students’ risk, ambiguity, and prosocial preferences. The risk and ambiguity tasks were carried out using an online laboratory experiment with oTree [31], while the donation task was conducted in an online field setting. These measures aimed to capture behavioral traits that might influence subsequent health decisions.

##### The risk task

This study employed the certainty equivalent method to measure individual risk preferences [32–34]. Participants were presented with nine pairs of choices between risky lotteries (left column) and fixed monetary amounts (right column). The lottery payouts had known probabilities of a 50% chance of winning 9 yuan and a 50% chance of winning 3 yuan. The fixed amounts in the right column ranged from 3 to 9 yuan, increasing by 0.75 yuan per row. For each participant, one pair of options was randomly selected to determine the experimental payoff based on their choice. The switching row number, ranging from 1 to 10 (with 10 indicating never switching to the right column), reflects the participant’s levels of risk-taking, whereas higher values indicate greater levels of risk-taking.

##### The ambiguity task

This task was designed to measure individual preferences for ambiguity [34, 35]. The design was similar to the risk task, with the key distinction that the probabilities of the lotteries were unknown, thus creating ambiguity. Participants’ switching behavior between ambiguous and certain options was used to infer their ambiguity preference. Similarly, the switching row number, ranging from 1 to 10 (with 10 indicating never switching to the right column), reflects the participant’s levels of ambiguity preference, whereas higher values indicate greater levels of ambiguity tolerance.

##### The donation task

Prosocial preference was measured using a donation task. Participants received a fixed endowment of 50 yuan as compensation for participating in the online survey. They were then asked to decide how much of this endowment they wished to donate to the Tencent Charity project “Million Breaths for ALS (Amyotrophic Lateral Sclerosis),” with the remaining amount being their personal payoff [36–38]. The donation task was based on the design of [39] and served as an incentive-compatible measure of prosocial preference, providing insight into participants’ willingness to sacrifice personal monetary gains for the benefit of others.

We next introduce the vaccination data, which constitutes the second data component of this robustness check. Starting on 16 March 2021, Wuhan University conducted a mass vaccination campaign over three days, using the campus stadium as the central vaccination site. To gather information on vaccination status, a follow-up survey of participants from previous behavioral preference experiments was conducted between 20 March and 12 April^25^.

This survey asked participants whether they had received the initial dose of the COVID-19 vaccine and required those vaccinated to provide a screenshot of their vaccination status from Wuhan University’s official vaccination appointment system. For those who had not been vaccinated, the survey probed the reasons for non-vaccination.

On average, participants took 3 to 4 minutes to complete each questionnaire, and they received a reward of 3 to 8 yuan (about 0.42 to 1.13 dollars) for their participation. Ultimately, complete data were collected from 296 participants in the behavioral preferences experiment.

#### Data

Table 7 provides a summary of the variables collected in the Student Sample. Among the 296 participants, 178 voluntarily received the initial dose of the COVID-19 vaccine within one month, representing 60% of the sample (See Panel A). For those who had not been vaccinated, the reasons were classified into two categories: participants who indicated they did not plan to get vaccinated or did not want to be vaccinated were classified as “Not vaccinated/do not plan to get vaccinated”, with a non-vaccination rate of 17% (51 participants). Those who cited reasons such as lack of time or physical conditions were classified as “May get vaccinated,” comprising 23% (67 participants) of the unvaccinated group.

**Table 7:** Data summary of the Student Sample

Variable name	Variable explanation	Mean	Sd
*Panel A: The dependent variables*			
Vaccinated	Already vaccinated = 1; Not vaccinated = 0	0.60	0.49
Vaccination status	Not vaccinated/do not plan to get vaccinated = 0; May get vaccinated = 1; Already vaccinated = 2	1.43	0.77
*Panel B: Key independent variables*			
Prosocial preference	The donation amount in the donation task (0–50)	23.95	15.70
Risk taking	The switching point in the risk task (ranging from 1 to 10), where higher values indicate greater levels of risk-taking.	4.99	0.98
Ambiguity preference	The switching point in the ambiguity task (ranging from 1 to 10), where higher values indicate higher levels of ambiguity tolerance.	4.61	1.15
*Panel C: Control variables*			
Age	Age of participants	18.69	0.91
Male	Male = 1; Female = 0	0.43	0.50
Major	Medical major = 1; Other majors = 0	0.53	0.50
Wuhan	Wuhan = 1; Other cities = 0	0.04	0.20
Family Covid infection	Family members infected = 1; Otherwise = 0	0.01	0.08
Healthcare knowledge (1–5)	Knowledge level of healthcare industry, very unfamiliar = 1, very familiar = 5	3.13	1.01

Panel B in Table 7 presents the key behavioral preferences measured in the preliminary experiments. The average donation amount in the prosocial task was 23.95 yuan, with a donation rate of 47.9%, indicating prosocial behavior consistent with previous experimental studies [28]. The risk and ambiguity tasks indicated general risk-averse and ambiguity-averse behavior among participants, with average scores of 4.99 and 4.61, respectively. These findings align with Shachat et al. [34], reporting similar average scores (4.95 and 4.47) under the same experimental design in March 2020.

Panel C in Table 7 includes other control variables relevant to the analysis. In the dataset, 43% of participants were male, and the average age was approximately 19 years. A small proportion (1%) had family members previously infected with COVID-19. Of the participants, 53% were from medical majors, and 4% were from Wuhan. Participants’ knowledge of the healthcare industry was assessed through self-reported familiarity, ranging from 1 (very unfamiliar) to 5 (very familiar), with an average score of 3.13.

#### Results

To examine the factors that influence the vaccination behavior of college students, we used the actual vaccination status of the participants as the dependent variable. The analysis controlled for individual-level factors, including age, gender, major, and hometown (Wuhan vs. other locations), as well as session-fixed effects, family COVID-19 infection experience, and knowledge level of the healthcare industry. The key explanatory variables were prosocial, risk, and ambiguity preferences elicited in the earlier experiment. Table 8 presents average marginal effects estimated from logit and ordered logit models. Columns (1)–(2) report effects on the binary outcome of being vaccinated. Columns (3)–(5) and (6)–(8) display marginal effects from two separate ordered logit models (Models 3 and 4), corresponding to the probabilities of being unvaccinated, undecided, and vaccinated, respectively.^26^

**Table 8:** Vaccination and preferences (college students)

	Logit		Ordered logit (Model 3)			Ordered logit (Model 4)		
Dependent variable	Vaccinated		Vaccination status			Vaccination status		
			Unvax	Undecided	Vax	Unvax	Undecided	Vax
	(1)	(2)	(3)	(4)	(5)	(6)	(7)	(8)
Prosocial preference (0–50)	0.003*	0.004*	−0.003**	−0.002**	0.004**	−0.003**	−0.002**	0.004**
	(0.002)	(0.002)	(0.001)	(0.001)	(0.002)	(0.001)	(0.001)	(0.002)
Risk taking (1–10)	0.016	0.031	−0.011	−0.007	0.017	−0.017	−0.011	0.028
	(0.028)	(0.029)	(0.017)	(0.011)	(0.028)	(0.018)	(0.011)	(0.029)
Ambiguity preference (1–10)		−0.033				0.014	0.009	−0.023
		(0.027)				(0.016)	(0.010)	(0.026)
Session fixed effect	Yes	Yes	Yes			Yes		
Control variables	Yes	Yes	Yes			Yes		
Model $\chi ^2$	13.175	13.910	16.551			17.410		
Observations	296	296	296			296		

1. This table reports average marginal effects from logit and ordered logit models. Columns (1)–(2) present effects on the binary outcome of being vaccinated. Columns (3)–(5) (Model 3) and (6)–(8) (Model 4) report marginal effects on the probabilities of being unvaccinated, undecided, and vaccinated, respectively, based on ordered logit specifications. Robust standard errors are shown in parentheses. ***, **, and * indicate statistical significance at the 1%, 5%, and 10% levels, respectively

2. Unvax = not vaccinated and do not plan to get vaccinated ($Vaccination\ status=0$); Undecided = may get vaccinated ($Vaccination\ status=1$); Vax = already vaccinated ($Vaccination\ status=2$)

3. Control variables include age, gender, major, Wuhan hometown, family COVID infection history, and healthcare knowledge (see Panel C in Table 7)

The results in Table 8 indicate that prosocial preference is a significant predictor of vaccination behavior among college students, suggesting that those with higher prosocial preference were more likely to receive the vaccine. The marginal effects suggest that a one-unit increase in the donation amount raises the probability of being vaccinated by 0.4 percentage points in Column (2). In Model 4, the same increase raises the probability of being already vaccinated by 0.4 percentage points, while reducing the probabilities of being not vaccinated and may get vaccinated by 0.3 and 0.2 percentage points, respectively. This finding is consistent with Hypothesis 1a, which predicts that greater prosocial preference increases self-vaccination intensity. While the coefficient on risk taking is positive—implying that more risk-seeking individuals are more likely to vaccinate—the estimate is not statistically significant. This result provides no evidence in support of Hypothesis 1b, which predicts that adults’ risk-taking tendencies are associated with their vaccination decisions. In contrast, ambiguity preference does not show a statistically significant association with vaccination behavior, suggesting that aversion to ambiguity plays a limited role in shaping vaccination choices in this context.

These findings imply that the influence of risk and ambiguity preferences on vaccination is limited. A potential explanation is that vaccination campaigns are an activity with a collective nature and under social norm pressures, making prosocial preference play a more important role than other individual preferences, such as risk and ambiguity preferences. The robustness of prosocial preference as a key determinant is further demonstrated by the consistent findings across different model specifications. The predictive power of experimentally measured prosocial preference several months before underscores the value of behavioral traits in predicting health-related behaviors over time.

### Children vaccination: alternative children vaccination definition

To address concerns about the dependent variable used in our main analysis of Wave 2, we conduct a robustness check in which the outcome is redefined using an alternative criterion. The baseline definition assigns a value of 1 only if all children aged 3 or above within a household have received the vaccine, reflecting our interest in whether parents choose to vaccinate all of their eligible children. This stricter criterion captures a stronger form of household-level vaccine acceptance, which is especially relevant from a public health perspective aiming for herd immunity.

In this robustness check, we adopt a less restrictive outcome definition, coding the variable as 1 if at least one child aged 3 or above in the household was vaccinated. This alternative definition allows us to assess whether the observed effects of behavioral preferences—particularly risk taking and omission bias—remain consistent when considering partial rather than more complete child vaccination within families. As shown in Table 9, the results closely mirror those in the main analysis (Table 6), thereby strengthening the credibility of our findings.

**Table 9:** Parental behavioral preferences and children vaccination

Dependent variable	At least one child vaccinated (age $\ge 3$)		
	(1)	(2)	(3)
Prosocial preference	−0.004	−0.023	−0.024
	(0.018)	(0.019)	(0.019)
Risk taking	0.049*	0.053*	0.050*
	(0.028)	(0.028)	(0.028)
Omission bias		−0.080***	−0.081***
		(0.014)	(0.014)
Control for characteristics	Yes	Yes	Yes
Control wave fixed effect	Yes	Yes	Yes
Control city fixed effect	No	No	Yes
Model $\chi ^2$	30.556***	57.081***	57.014***
Observations	706	706	706

1. This table reports marginal effects from the logit model, with robust standard errors in parentheses. ***, **, and * indicate statistical significance at the 1%, 5%, and 10% levels, respectively

2. Control for characteristics: Age, gender, education, work in the healthcare industry, annual income, CPC

## Conclusion

This study investigates how individual behavioral preferences influence COVID-19 vaccination decisions, both for oneself and for one’s children. Drawing on large-scale public surveys and an auxiliary incentivized experiment, we find that prosocial preference positively predicts adults’ vaccination behavior, while it has no significant effect on parental decisions to vaccinate their children. In contrast, parents’ willingness to take risks is positively associated with childhood vaccination uptake but has limited influence on adult vaccination. Additionally, omission bias emerges as a key behavioral factor contributing to parental vaccine hesitancy. These findings highlight that distinct behavioral mechanisms underlie self-directed versus child-directed vaccination decisions.

Our findings speak to several active debates in behavioral health economics. First, our study confirms that prosocial preferences are important drivers of adult vaccination. This finding extends Campos-Mercade et al. [12], which focuses on the role of prosociality in preventive health behaviors other than vaccination. Moreover, whereas Campos-Mercade et al. [40] demonstrate external economic incentives, our analysis centers on internal behavioral motivators—such as prosociality, risk preference, and omission bias. In practice, combining both intrinsic and extrinsic mechanisms may offer a more effective strategy for reducing vaccine hesitancy. Second, while studies such as Andersson et al. [41] demonstrate how positive vaccine information can shape situational beliefs and reduce protective behaviors, our findings emphasize the stable influence of internal behavioral traits such as prosocial preference, risk preference, and omission bias. Third, to interpret why some parents vaccinate themselves but hesitate to vaccinate their children, we consider economic theories of parental altruism as one possible explanatory lens. While our data do not directly measure parenting styles, frameworks such as paternalistic versus Beckerian altruism [42, 43] suggest that parents who prioritize autonomy may make different decisions for their children than for themselves. Such theoretical distinctions help illuminate why behavioral drivers of vaccination may diverge across adult–child contexts, particularly under uncertainty or perceived medical risk.

These insights have several policy implications. First, the observed association between prosociality and adult vaccination points to a potential behavioral strategy for increasing vaccine uptake. Messaging strategies that emphasize societal benefits—such as herd immunity and community protection—may help activate individuals’ prosocial preferences, thereby encouraging vaccination. This interpretation is consistent with prior experimental evidence [44, 45]. Second, to promote childhood vaccination, policymakers should recognize the psychological hurdles posed by omission bias and risk aversion. Communication strategies that transparently present both vaccine benefits and risks—while addressing parents’ concerns about side effects—could mitigate behavioral resistance. Finally, generating reliable behavioral evidence to inform public health decisions requires methodological rigor. Our study demonstrates the practical potential of combining survey data with incentivized experiments to produce robust insights that can inform vaccine policy design.

Despite these contributions, several limitations should be acknowledged. Our study examines how prosocial preferences and risk attitudes shape COVID-19 vaccination decisions for oneself and one’s children. While our design allows for rich behavioral insights and strong internal validity, it is important to recognize that vaccination choices were made within a specific institutional context. Although vaccination was officially voluntary in China, and no formal access restrictions were imposed in the surveyed cities during the study periods^27^ , individuals may still have perceived indirect incentives, such as smoother travel or organizational compliance. These perceptions may obscure the line between intrinsic preferences and responses to institutional cues, meaning that vaccination status may not fully reflect underlying behavioral traits. This limits causal interpretation and generalizability.

This study was not pre-registered, and we acknowledge this as a limitation of our research design. Nonetheless, the study was designed to provide a transparent evaluation of how behavioral preferences influence actual decisions to receive COVID-19 vaccinations. To mitigate concerns about reproducibility, we conducted multiple robustness checks during the data analysis. All data, materials, and code necessary to replicate the experimental tasks and empirical results are available in the project repository on the Open Science Framework^28^.

Future research could build on these findings in several directions. First, to address concerns about institutional confounding, future studies should incorporate more direct measures of perceived constraints and motivations—such as beliefs about access requirements, workplace norms, or travel restrictions—that may influence vaccination behavior independently of behavioral traits. Second, it would be informative to examine how stable behavioral preferences, such as risk aversion or omission bias, interact with shifting policy environments. For example, are risk-averse individuals more responsive to mandatory vaccination policies, or does omission bias become more salient when public discourse emphasizes vaccine side effects? Studying such interactions across different phases of a public health crisis could reveal how institutional design shapes the effectiveness of behavioral interventions. Third, cross-country comparisons that leverage institutional variation—such as differences in mandate strength, communication strategies, or levels of public trust—can help isolate culturally contingent versus universal drivers of vaccine hesitancy. Finally, linking experimentally or survey-elicited behavioral traits to administrative vaccination records would improve measurement precision and allow for better modeling of the pathways from psychological disposition to concrete health actions.

## Supplementary Information

Supplementary Material 1.

## Data Availability

All data, materials, and code necessary to replicate the experimental tasks and empirical results are available in the project repository on the Open Science Framework https://osf.io/nc7th/.

## Footnotes

1 See the World Health Organization (WHO) website. Link: https://www.who.int/news-room/facts-in-pictures/detail/immunization, accessed February 11, 2024.

2 See the World Health Organization (WHO) website. Link: https://www.who.int/zh/news-room/spotlight/ten-threats-to-global-health-in-2019, accessed February 11, 2024.

3 Among them, 167 respondents participated in both Wave 1 and Wave 2 data collection.

4 While not fully nationally representative, our sample includes parenting-age adults from major cities—groups critical for understanding vaccination decisions and urban health risks. We refer to these samples as the “general public” for simplicity and to distinguish them from the student sample used in robustness checks.

5 This study was not pre-registered; however, the research objective was clearly defined ex ante as an investigation into how behavioral preferences influence actual decisions to receive COVID-19 vaccinations. We further elaborate on this point in the Conclusion section.

6 Wenjuanxing (www.wjx.cn) is a leading survey platform in China, offering similar functionalities to Amazon Mechanical Turk and Qualtrics. Numerous studies, such as Bao et al. [46], Jain et al. [47], and Zhang et al. [48], have used this platform for data collection. In addition to the compensation received by the participants, the survey platform charges fees based on the difficulty of reaching the research targets and the length of the questionnaire.

7 Hefei, Wuhan, Zhengzhou, and Xi’an are all provincial capitals in China, each located in distinct regions: Hefei in the east, Wuhan in central China, Zhengzhou in the north-central, and Xi’an in the west. The sample from these four cities, given their geographical distribution and size, is quite representative of China with regard to the research question.

8 The screening process for the questionnaire involved trap questions, which included common knowledge or attention checks, inserted randomly to evaluate respondent attentiveness and sincerity. Responses that failed the trap questions were considered invalid. Each participant could complete the survey only once, and only those who provided valid responses were eligible for the reward.

9 See AppendixE.1 in Supplementary Material for prosocial preference questions.

10 Questions concerning sexual behavior, drug abuse, and alcohol consumption were excluded as these topics were considered too sensitive for Chinese participants and thus restricted by the survey platform. See Appendix E.2 in Supplementary Material for DOSPERT risk preference questions.

11 Appendix B in Supplementary Material provides detailed variable definitions and summary statistics. Table B1 presents complete definitions and summary statistics for all measured variables. Table B2 compares the demographic characteristics of our sample with national population statistics, including age, gender, income, and healthcare employment. The sample tends to be younger and more affluent, possibly due to the use of an online survey platform and the sampling focus on four provincial capitals (Wuhan, Zhengzhou, Hefei, and Xi’an).

12 It is worth noting that, due to the online nature of the questionnaire, the ages of the participants were slightly lower than the average age of the population in each city.

13 As a robustness check, we present the Linear Probability Model (LPM) regression results in Table C1, located in Supplementary Material Appendix C.1. The estimates remain statistically significant and consistent with our main findings in most specifications, with the exception of Column (5), where the result becomes insignificant, likely due to sample size constraints.

14 We use age 55 as the primary cutoff to define older individuals, as it was the statutory retirement age for female employees in China at the time of the survey. Age 45 is also used as an alternative threshold to capture middle-aged and older respondents, in line with WHO classifications.

15 To ensure temporal consistency with Wave 1 (surveyed in May 2021), we use retrospective information collected in Wave 2 (conducted in December 2021–January 2022) on the timing of respondents’ first vaccine dose. Specifically, the outcome variable is defined based on whether the individual had received their first dose by the end of May 2021, allowing comparability across waves. Matched respondents across waves are only included once in the pooled analysis to avoid duplication.

16 The follow-up survey in Wave 2 was conducted through the platform’s sample service. All 1,268 participants from Wave 1 were re-invited using user ID-based targeting within the platform. Since we did not have access to participants’ personal contact information, all follow-up communication and response matching were carried out by the platform’s internal system. Matching across waves relied on consistent internal user IDs, ensuring accurate linkage between datasets. Of those invited, only 213 completed the follow-up questionnaire, yielding a recontact rate of 16.8%. Among these, 167 respondents met the criteria for the vaccine module analysis, specifically having at least one child over the age of three. To increase the sample size for the Wave 2 analysis, we additionally recruited new participants through the same platform, resulting in 539 valid responses from parents of children aged three or older.

17 See Appendix E.3 in Supplementary Material for detailed questions.

18 China has approved the use of COVID-19 vaccines for individuals aged 3 and older (with no upper age limit, the lower age limit has gradually been relaxed from 18 years to 12 years and then to 3 years).

19 This definition reflects our interest in whether parents choose to vaccinate all of their eligible children, rather than just some. Given that herd immunity depends on achieving high overall coverage, this distinction is important. From a policy perspective, it shifts the focus from encouraging minimal uptake to promoting complete vaccination within families, which is more consistent with public health objectives.

20 Appendix C.4 in Supplementary Material provides exploratory evidence on intra-household discrepancies between parents’ and children’s vaccination decisions.

21 As a robustness check, we report the LPM regression results in Table C2, located in Supplementary Material Appendix C.1. The estimates are statistically significant and consistent with our main findings.

22 As a robustness check, we use an alternative specification in Table 9 (see Children vaccination: alternative children vaccination definition section), where the dependent variable “At least one child vaccinated (age $\ge 3$)” is defined as 1 if at least one child aged 3 or above within the household has received the vaccine. The results are robust to this alternative definition.

23 To assess potential selection bias, we compared the baseline characteristics of followed-up and non-followed-up respondents from Wave 1 (Table C4). While some observable differences exist, prosocial preference—the key variable of interest—did not differ significantly between the groups. To further assess robustness, we conducted separate regressions using the Wave 1 follow-up sample and the full Wave 2 sample (Table C5). The results remain consistent, suggesting that our findings are not driven by selection bias.

24 Since Wuhan University students were among the first in China to receive widespread vaccination, limited information was available on the COVID-19 vaccine’s side effects among university students at that time. No targeted intervention or promotional campaign was conducted, resulting in relatively uncontaminated behavioral influences.

25 Since women on their menstrual period were ineligible to receive the vaccine at that time, the data collection period spanned nearly a month, effectively mitigating the influence of menstrual factors on female vaccination rates.

26 As a robustness check, we report the Linear Probability Model (LPM) regression results in Table C3, located in Supplementary Material Appendix C.1. The estimates are statistically significant and consistent with our main findings.

27 For additional details on China’s vaccination policies and access regulations during the survey periods, please refer to Appendix D in Supplementary Material.

28 https://osf.io/nc7th/.
