# Supplementary material for: Vaccination for adults and their children: insights from survey and experimental data
Source: Health Econ Rev. 2025 Oct 24;15:89. doi: 10.1186/s13561-025-00685-w (PMC12553180; doi:10.1186/s13561-025-00685-w)
Supplement: Supplementary file 1 — Supplementary Material 1. [file 13561_2025_685_MOESM1_ESM.pdf]

Appendix

A Summary of data collection

Table A1: Summary of data collection waves

Subjects	Obs.	Location	Task type
**Wave 1: Vaccination of adults (May 2021)**			
The public	1268	Wuhan, Hefei, Zhengzhou, Xi'an	Online survey with a fixed reward
**Wave 2: Vaccination of children (December 2021 - January 2022)**			
The public	706	Wuhan, Hefei, Zhengzhou, Xi'an	Online survey with a fixed reward
**Student Sample: Vaccination of college students (March-April 2021)**			
Students	296	Wuhan University	Online experiment with incentive rewards

B Variable definitions and summary statistics

B.1 Variable definitions and summary statistics for all samples

Table B1: Variable definitions and summary statistics for all samples

Variable name	Variable explanation	Mean	Sd
Panel A. Public sample (own vaccination) ($n = 1268$)			
Received initial vaccine dose	Whether received the initial vaccine dose by June 2, 2021 (yes=1; no=0)	0.74	0.44
Prosocial preference (1-5)	Level of prosocial preference (1=lowest; 5=highest)	3.91	0.71
Risk taking (1-5)	Level of risk-taking preference (1=lowest; 5=highest)	2.88	0.52
Age	Age of the participants (years)	30.62	9.88
Male	Gender of participants (male=1; female=0)	0.53	0.50
Work in the healthcare industry	Whether the participant works in the healthcare industry (yes=1; no=0)	0.02	0.14
CPC (Communist Party of China) membership	Membership of the CPC (yes=1; no=0)	0.27	0.44
Have travel plans in the near future	Have travel plans in near future (yes=1; no=0)	0.26	0.44
Panel B. Public sample (children's vaccination) ($n = 706$)			
All child vaccinated (age $\geq$ 3)	Whether all children aged 3 or above are vaccinated (yes=1; no=0)	0.82	0.38
Prosocial preference (1-5)	Level of prosocial preference (1=lowest; 5=highest)	4.04	0.72
Risk taking (1-5)	Level of risk-taking preference (1=lowest; 5=highest)	2.91	0.54
Omission bias (1-7)	Tendency to avoid action due to omission bias (1=lowest; 7=highest)	3.01	0.91
The number of children	Total number of children the participant has	1.37	0.57
Age	Age of the participant (years)	36.73	9.14
Male	Gender of the participant (male=1; female=0)	0.47	0.50
Work in the healthcare industry	Whether the participant works in the healthcare industry (yes=1; no=0)	0.04	0.19
CPC (Communist Party of China) membership	Whether the participant is a CPC member (yes=1; no=0)	0.30	0.46
Parents have been vaccinated	Whether the participant (parent) has been vaccinated (yes=1; no=0)	0.98	0.15
Panel C. Student participants ($n = 296$)			

Continued on next page

Table B1 (Continued)

Variable name	Variable explanation	Mean	Sd
Vaccinated	Intended to reduce personal protective measures after receiving the COVID-19 vaccine (yes = 0; no = 2; Not Sure = 1)	0.60	0.49
Vaccination status	Not vaccinated/no plan=0; May vaccinate=1; Vaccinated=2	1.43	0.77
Prosocial preference (0-50)	The donation amount in the donation task (higher=more prosocial)	23.95	15.70
Risk taking (1-10)	The switching point in the risk task (higher=more risk-taking)	4.99	0.98
Ambiguity preference (1-10)	The switching point in the ambiguity task (higher=more tolerant)	4.61	1.15
Age	Age of the participants (years)	18.69	0.91
Male	Gender of the participant (male=1; female=0)	0.43	0.50
Major	Major of the participant (Medical=1; Others=0)	0.53	0.50
Wuhan	Whether the participant lives in Wuhan (yes=1; no=0)	0.04	0.20
Family Covid infection	Family had Covid-19 (yes=1; no=0)	0.01	0.08
Healthcare knowledge (1-5)	Familiarity with healthcare (1=very unfamiliar; 5=very familiar)	3.13	1.01

Notes: Categorical variables such as income and edu are not included in this table because their values are not meaningfully summarized using means or standard deviations. Instead, the proportions across different categories for these variables are reported in the main text.

B.2 Sample vs. National Demographics

Table B2: Comparison of Sample and National Demographics

	Wave 1	Wave 2	2021 National Demographics
Age	30.62	29.29	38.8
Male (%)	52.52	46.08	51.19
Work in the healthcare industry (%)	2.05	4.28	0.99
Annual income (Yuan)	99787	105966	80237

Notes:

1. National demographic statistics on gender and annual income are based on the 2021 China Statistical Yearbook. Age distribution data are obtained from the Seventh National Population Census (2021). Data on healthcare employment are sourced from the official website of the Chinese central government.

2. Annual income is measured in categories. To approximate the average income, we assign representative values to each group: 20,000 for category 1 ($< 20K$), 60,000 for category 2 ($20K - 100K$), 200,000 for category 3 ($100K - 300K$), 650,000 for category 4 ($300K - 1M$), and 1,000,000 for category 5 ($> 1M$).

C Additional Analysis

C.1 Robustness Check: Linear Probability Model

Table C1: Vaccination and behavioral preferences (adults)

	Linear Probability Model				
Dependent variable	Received the initial dose of the COVID-19 vaccine				
Data	All sample			Above 45 years	Above 55 years
	(1)	(2)	(3)	(4)	(5)
Prosocial preference	0.089***	0.088***	0.085***	0.123***	0.121
	(0.018)	(0.018)	(0.018)	(0.038)	(0.074)
Risk taking	0.037	0.026	0.021	0.003	-0.073
	(0.024)	(0.024)	(0.024)	(0.043)	(0.084)
Constant	0.048	0.076	0.118	0.170	0.709
	(0.115)	(0.115)	(0.117)	(0.332)	(0.934)
Control for characteristics	Yes	Yes	Yes	Yes	Yes
Have travel plans in the near future	No	Yes	Yes	Yes	Yes
Control city fixed effect	No	No	Yes	Yes	Yes
Control questionnaire effect				Yes	Yes
Observations	1268	1268	1268	264	73

Notes:

1. Robust standard errors are reported in parentheses; ***, **, and * indicate statistical significance at the 1%, 5%, and 10% levels, respectively.
2. Control for characteristics: Age, gender, education, work in the healthcare industry, annual income, CPC.
3. Questionnaire Effect: Columns (1)–(3) use data from wave 1. However, since columns (4) and (5) require sample filtering based on age, the wave 1 sample size is relatively small. Therefore, we also include samples from wave 2. Although Wave 2 was conducted later, we use self-reported vaccination dates to ensure that the outcome variable consistently reflects vaccination status as of May 2021. To account for potential differences arising from the use of different questionnaires, we control for questionnaire-fixed effects in the regression models for columns (4) and (5).

Table C2: Parental behavioral preferences and children vaccination

	Linear Probability Model		
Dependent variable	All child vaccinated (age $\geq$ 3)		
	(1)	(2)	(3)
Prosocial preference	0.013	-0.002	-0.003
	(0.021)	(0.020)	(0.020)
Risk taking	0.053**	0.057**	0.058**
	(0.026)	(0.026)	(0.026)
Omission bias		-0.081***	-0.081***
		(0.016)	(0.016)
Constant	0.376***	0.715***	0.698***
	(0.133)	(0.139)	(0.144)
Control for characteristics	Yes	Yes	Yes
Control wave fixed effect	Yes	Yes	Yes
Control city fixed effect	No	No	Yes
Observations	706	706	706

Notes:

1. Robust standard errors are reported in parentheses; ***, **, and * indicate statistical significance at the 1%, 5%, and 10% levels, respectively.
2. Control for characteristics: Age, gender, education, work in the healthcare industry, annual income, CPC.
3. Wave fixed Effect: The analysis includes both follow-up respondents from Wave 1 and newly recruited participants in Wave 2; wave fixed effects are included to account for systematic differences across waves.

Table C3: Vaccination and preferences (college students)

	Linear Probability Model	
Dependent variable	Vaccinated	
	(1)	(2)
Prosocial preference (0-50)	0.003*	0.004*
	(0.002)	(0.002)
Risk taking (1-10)	0.017	0.032
	(0.029)	(0.030)
Ambiguity preference (1-10)		-0.033
		(0.027)
Constant	0.555	0.605
	(0.629)	(0.630)
Session fixed effect	Yes	Yes
Control variables	Yes	Yes
Observations	296	296

Notes:

1. Robust standard errors are reported in parentheses; ***, **, and * indicate statistical significance at the 1%, 5%, and 10% levels, respectively.
2. Control variables: Age, gender, major, wuhan, family covid infection, healthcare knowledge.

C.2 Robustness Check: Parental Preferences and Children's Vaccination Across Waves

Table C4: Comparison of Followed-up and Non-Followed-up Respondents in Wave 2

	Not Followed-up	Followed-up	q-value
Received the initial dose of the vaccine (June 2, 2021)	1.00	0.97	< 0.01
	(0.00)	(0.17)	
Prosocial preference (1–5)	3.89	3.98	0.88
	(0.71)	(0.71)	
Risk taking (1–5)	2.86	2.98	0.02
	(0.53)	(0.46)	
Age	29.77	34.82	< 0.01
	(10.20)	(6.73)	
Male (%)	53.84	46.01	0.37
	(49.88)	(49.96)	
Education	3.51	3.77	< 0.01
	(0.89)	(0.76)	
Work in the healthcare industry (%)	2.18	1.41	1.00
	(14.61)	(11.81)	
Annual income	1.93	2.52	< 0.01
	(0.79)	(0.64)	
CPC (Communist Party of China) membership (%)	25.40	32.39	0.35
	(43.55)	(46.91)	
Have travel plans in the near future (%)	23.89	37.09	< 0.01
	(42.66)	(48.42)	
Observations	1055	213	

Note: Multiple hypothesis testing adjusted False Discovery Rate (FDR) q-values (10 comparisons) based on two-sided Wilcoxon rank-sum tests.

Table C5: Parental behavioral preferences and children vaccination

Dependent variable	All child vaccinated (age $\geq$ 3)					
Data	Wave 1 Follow-up			Wave 2		
	(1)	(2)	(3)	(4)	(5)	(6)
Prosocial preference	0.026	-0.045	-0.051	0.007	-0.001	-0.000
	(0.050)	(0.050)	(0.048)	(0.021)	(0.022)	(0.022)
Risk taking	0.105*	0.089	0.092*	0.053	0.059*	0.059*
	(0.061)	(0.057)	(0.055)	(0.034)	(0.034)	(0.034)
Omission bias		-0.158***	-0.163***		-0.056***	-0.055***
		(0.034)	(0.031)		(0.017)	(0.017)
Control for characteristics	Yes	Yes	Yes	Yes	Yes	Yes
Control city fixed effect	No	No	Yes	No	No	Yes
Model χ^2	18.35**	27.06***	28.74***	33.92***	32.39***	44.17***
Observations	167	167	167	539	539	539

Notes:

1. This table reports marginal effects from the logit model, with robust standard errors in parentheses. ***, **, and * indicate statistical significance at the 1%, 5%, and 10% levels, respectively.
2. Control for characteristics: Age, gender, education, work in the healthcare industry, annual income, CPC.
3. Data source: Columns (1)–(3) use follow-up data from Wave 1 respondents collected during Wave 2; Columns (4)–(6) use independent observations from the main Wave 2 sample.

C.3 Validate the correlation of behavioral preference data

The main empirical analysis is based on two waves of public survey data, in which prosocial and risk preferences were measured using self-reported subjective questionnaires among a broad population sample. In contrast, the Student Sample employed incentivized experimental tasks to elicit behavioral preferences.

To ensure the comparability and relevance of the research conclusions drawn from both public and student samples,[29] we analyzed the correlations between non-incentivized and incentivized[30] assessments of risk-taking and prosocial behaviors using participant data from four cities. The correlation analysis involved a total of 1,133 additional valid samples. Panel A of Table C6 provides comprehensive descriptive statistics on basic demographic information for these 1,133 samples, which can be compared to those of the previous samples detailed in Table 1.

The first four rows in Panel B of Table C6 show the mean and standard deviation for both incentivized and non-incentivized measures of risk and prosocial preferences. The fifth row demonstrates the positive correlation between incentivized and non-incentivized measures for risk taking and prosocial preference, significant at 5% and 1% levels, respectively. These correlations, while modest, provide some support for the external relevance of non-incentivized preference measures in broader population samples.

[29]The paper received feedback highlighting that preference measurements during the first and second waves lacked incentive-based methods. Consequently, we undertook additional data collection, capturing both incentivized and non-incentivized behavioral preferences, and examined the relationship between these sets of data.

[30]The incentivized prosocial task corresponds to the dictator game with the initial endowment of 5 yuan RMB, while the incentivized risk task is the same as the Student Sample.

Table C6: Correlation between incentivized and non-incentivized preferences

Panel A: Descriptive statistics		
	Mean	Sd
Age	30.58	9.99
Male (%)	46.95	49.93
Education[1]	3.38	1.04
Work in the healthcare industry (%)	3.97	19.54
Annual income[2]	2.00	0.86
CPC (Communist Party of China) membership (%)	23.65	42.51
Have travel plans in the near future (%)	24.18	42.84
Panel B: Correlation analysis		
	Risk taking	Prosocial preference
Mean (Incentivized)	4.37	2.73
Sd (Incentivized)	1.86	1.24
Mean (Non-incentivized)	2.87	3.95
Sd (Non-incentivized)	0.54	0.75
Pairwise correlations[3]	0.09**	0.10***
p-value	0.02	< 0.01
Observations[4]	727	1133

Notes:

1. Education: 1 = didn't attend high school; 2 = High school or similar; 3 = College; 4 = Bachelor's degree; 5 = Master's degree or above.
2. Annual income: 1 = Below 20K (20,000 RMB); 2 = 20K to 100K; 3 = 100 K to 300 K; 4 = 300 K to 1 M; 5 = Above 1 M.
3. ***, **, and * indicate statistical significance at the 1%, 5%, and 10% levels, respectively.
4. Because certain participant responses displayed several jump points in the incentivized risk task, those specific samples were discarded from the initial collection. Consequently, only 727 out of 1133 samples were included in the final correlation analysis concerning risk taking.

C.4 Intra-household Vaccination Discrepancy

In this section, we provide exploratory evidence on intra-household discrepancies between parents' and children's vaccination decisions. We estimate a logit model in which the dependent variable equals one if the parent is vaccinated but the child is not, and zero otherwise. Constructing this outcome requires information on both parental and child vaccination status. Parental vaccination status was collected in Wave 1 and child vaccination status in Wave 2. Due to re-contact limitations of the online survey platform, we were able to follow up with only 167 households from Wave 1 in Wave 2, yielding a restricted subsample in which both parental and child vaccination decisions are observed.

Table C7 reports marginal effects from the logit regressions. Risk taking is negatively associated with the probability of a discrepancy, indicating that more risk-taking parents are less likely to vaccinate only themselves. Omission bias is positively associated, implying that parents with stronger omission bias are more likely to vaccinate themselves while not vaccinating their child. Both patterns arise because risk taking increases, and omission bias decreases, the probability of child vaccination, which in turn affects the likelihood that only the parent is vaccinated. Prosocial preference, by contrast, does not exhibit a statistically significant effect.

Table C7: Determinants of Being Vaccinated Oneself but Not Vaccinating One's Child

Dependent variable	Parent-only vaccination		
	(1)	(2)	(3)
Prosocial preference	-0.013	0.057	0.067
	(0.051)	(0.051)	(0.049)
Risk taking	-0.109*	-0.093	-0.097*
	(0.061)	(0.057)	(0.055)
Omission bias		0.155***	0.162***
		(0.033)	(0.031)
Control for characteristics	Yes	Yes	Yes
Control city fixed effect	No	No	Yes
Model χ^2	16.141 ***	26.032 ***	30.016***
Observations	167	167	167

Notes:

1. This table reports marginal effects from the logit model, with robust standard errors in parentheses. ***, **, and * indicate statistical significance at the 1%, 5%, and 10% levels, respectively.
2. Control for characteristics: Age, education, work in the healthcare industry, annual income, CPC.

D Institutional Context of COVID-19 Vaccination and Public Access Restrictions

China's national policy on COVID-19 vaccination was founded on the principle of "informed, consented, and voluntary" participation.[31] Vaccination campaigns were primarily executed through public health communication and community mobilization, rather than by legal compulsion. Throughout the pandemic, the core regulatory instruments governing individual mobility and access to public venues were health code systems and nucleic acid test results, not vaccination status.

The overall institutional framework was non-coercive. Local authorities were only permitted to strongly encourage COVID-19 vaccination without imposing mandatory requirements. The government primarily relied on public communication campaigns and persuasive messaging rather than enforcement measures. For example, the city of Wuhan issued the Initiative for COVID-19 Vaccination in Wuhan, calling on residents to voluntarily get vaccinated as a civic responsibility.[32] However, a limited number of local jurisdictions briefly experimented with measures that conditioned access to certain public facilities on vaccination records. For instance, in mid-2021, Jinjiang (Fujian Province) issued administrative notices requiring adults to present vaccination certification to enter government buildings, hospitals, schools, and commercial venues.[33] These local directives, however, were quickly met with regulatory pushback from central authorities. On April 11, 2021, the State Council's Joint Prevention and Control Mechanism publicly criticized such practices as overly simplistic and inconsistent with national policy.[34] Further clarification was issued by the National Health Commission (NHC) in September 2021, stating that exclusion of unvaccinated individuals from schools, hospitals, or transit stations was inappropriate and in violation of the voluntary nature of vaccination.[35]

The health code system, central to China's pandemic governance, integrated multiple dimensions of individual status, including recent test results, mobility history, and—later in the pandemic—vaccination records. Access to public transportation and social infrastructure typically required a green health code, which did not necessarily reflect vaccination status.[36] Even after the introduction of a "gold-coded" variant indicating vaccination, functional access remained tied to test results and exposure risk rather than inoculation per se.[37]

Importantly, in the cities and time periods covered by our survey—Wuhan (March-April 2021, May-June 2021, December 2021-January 2022), Xi'an, Zhengzhou, and Hefei (May-June 2021 and December 2021–January 2022)—we found no formal restrictions on the unvaccinated that would materially affect access to public venues. For instance, Zhengzhou health authorities explicitly stated in July 2021 that unvaccinated status would not impair an individual's mobility or access.[38] Our discussion is restricted to the time periods during which our survey were conducted. Whether similar or more coercive vaccination policies were implemented after January 2022 falls outside the scope of this study.

This institutional setting is theoretically consequential. While individuals may have anticipated social or logis-

[31] See Central Commission for Discipline Inspection and National Supervisory Commission website. Link: https://www.ccdi.gov.cn/yaowen/202107/t20210716_246297.html, accessed June 20th, 2025.

[32] See the People's Government of Wuhan Municipality website. Link: https://www.wuhan.gov.cn/sy/whyw/202103/t20210328_1657556.shtml, accessed June 20th, 2025.

[33] See Quanzhou Evening News. Link: https://baijiahao.baidu.com/s?id=1705121849349412610, accessed June 20th, 2025.

[34] See the People's Government of Beijing Municipality website. Link: https://www.beijing.gov.cn/ywdt/zybwdt/202104/t20210412_2352395.html, accessed June 20th, 2025.

[35] See the State Council of the People's Republic of China website. Link: https://www.gov.cn/xinwen/2021-09/07/content_5636053.htm, accessed June 20th, 2025.

[36] See the Hubei Government Services website. Link: http://zwfw.hubei.gov.cn/webview/yqzq/healthCode.html, accessed June 20th, 2025.

[37] See CNR News. Link: https://baijiahao.baidu.com/s?id=1695077155184807295, accessed June 20th, 2025.

[38] See Administration for Market Regulation of Zhengzhou City. Link: https://amr.zhengzhou.gov.cn/xwfb/5156494.jhtml, accessed June 20th, 2025.

tical benefits from vaccination (e.g., smoother travel or workplace compliance), the absence of strict enforcement mechanisms implies that vaccination decisions were not shaped by imminent material penalties. Thus, in evaluating the determinants of vaccination behavior, it is appropriate to interpret observed choices as arising from heterogeneous combinations of prosocial preference, risk preference, and social motivations, rather than from coercion-induced compliance.

E Key Survey Contents

This appendix presents the core survey items used to measure behavioral preference variables analyzed in the main text, including prosocial preferences, risk preferences, and omission bias. The full questionnaire materials are available in the Online Supplementary Materials at the Open Science Framework: https://osf.io/nc7th/.

E.1 Prosocial preference questions

Read the following statements and, assuming you are in that scenario, assess the likelihood of your participation in the activity or manifestation of a certain behavior from 1 to 5, where 1 represents extremely unlikely and 5 represents extremely likely.

1. **I will donate blood.**
   Extremely unlikely (1) □—□—□—□—□ Extremely likely (5)
2. **I will participate in volunteer activities for the long term.**
   Extremely unlikely (1) □—□—□—□—□ Extremely likely (5)
3. **Donate money to those who cannot afford medical treatment due to illness.**
   Extremely unlikely (1) □—□—□—□—□ Extremely likely (5)
4. **Give up your seat for the elderly, weak, disabled, or pregnant on public transport or subway.**
   Extremely unlikely (1) □—□—□—□—□ Extremely likely (5)

E.2 DOSPERT risk taking questions

Read the following statements and, assuming you are in that scenario, assess the likelihood of your participation in the activity or manifestation of a certain behavior from 1 to 5, where 1 represents extremely unlikely and 5 represents extremely likely.

1. **Publicly acknowledging that your preferences differ from those of your friends.**
   Extremely unlikely (1) □—□—□—□—□ Extremely likely (5)
2. **Invest 20% of annual income in a fund with steady growth potential.**
   Extremely unlikely (1) □—□—□—□—□ Extremely likely (5)
3. **I disagree with my parents on some important issues.**
   Extremely unlikely (1) □—□—□—□—□ Extremely likely (5)
4. **Invest 10% of annual income in speculative high-risk stocks.**
   Extremely unlikely (1) □—□—□—□—□ Extremely likely (5)
5. **Choose a job that you enjoy rather than one with good reputation but makes you unhappy.**
   Extremely unlikely (1) □—□—□—□—□ Extremely likely (5)
6. **As a passenger, I don't automatically fasten my seatbelt when sitting in the front seat of a car.**
   Extremely unlikely (1) □—□—□—□—□ Extremely likely (5)
7. **Invest 20% of your annual income into a new business venture.**
   Extremely unlikely (1) □—□—□—□—□ Extremely likely (5)
8. **In social situations, defend the side you believe in but is not popular.**
   Extremely unlikely (1) □—□—□—□—□ Extremely likely (5)

9. **Reveal a friend's secret to others.**
Extremely unlikely (1) □—□—□—□—□ Extremely likely (5)
10. **Try bungee jumping at least once.**
Extremely unlikely (1) □—□—□—□—□ Extremely likely (5)
11. **If possible, try flying a small plane.**
Extremely unlikely (1) □—□—□—□—□ Extremely likely (5)
12. **Leave your family and move to a distant place.**
Extremely unlikely (1) □—□—□—□—□ Extremely likely (5)
13. **Start a new career around the age of thirty-five.**
Extremely unlikely (1) □—□—□—□—□ Extremely likely (5)

E.3 Omission bias questions

Please read the following statements carefully and indicate your level of agreement from 1 to 7, where 1 means completely disagree and 7 means completely agree.

1. **Not vaccinating my child could put them at risk of contracting the COVID-19 virus. (Rate your agreement level from 1 to 7, where 1 means completely disagree and 7 means completely agree)**
Completely disagree (1) □—□—□—□—□—□—□ Completely agree (7)
2. **If my child experiences adverse reactions due to receiving the COVID-19 vaccine, I feel I should bear some responsibility. (Rate your agreement level from 1 to 7, where 1 means completely disagree and 7 means completely agree)**
Completely disagree (1) □—□—□—□—□—□—□ Completely agree (7)
3. **If my child contracts COVID-19 due to not receiving the COVID-19 vaccine, I feel I should bear some responsibility. (Rate your agreement level from 1 to 7, where 1 means completely disagree and 7 means completely agree)**
Completely disagree (1) □—□—□—□—□—□—□ Completely agree (7)

Note: Questions 1 and 3 are reverse-scored.
